# Alterations in Adipokine Levels Are Associated with Human Perinatal Anxiety and Depression

**DOI:** 10.3390/jcm14124102

**Published:** 2025-06-10

**Authors:** Ignacio Camacho-Arroyo, Mónica Flores-Ramos, Ismael Mancilla-Herrera, Fausto Manuel Cruz-Coronel, Blanca Farfan-Labonne, Laura Elena Jiménez-Aquino, María del Pilar Meza-Rodríguez, Joselin Hernández-Ruiz, Philippe Leff-Gelman

**Affiliations:** 1 Unidad de Investigación en Reproducción Humana, Instituto Nacional de Perinatología-Facultad de Química, Universidad Nacional Autónoma de México, Ciudad de Mexico 04510, Mexico; camachoarroyo@gmail.com; 2 Instituto Nacional de Psiquiatría Ramón de la Fuente Muñiz, Ciudad de Mexico 14370, Mexico; flores_ramos@hotmail.com; 3 Instituto Nacional de Perinatología, Ciudad de Mexico 11000, Mexico; mahi.25803@gmail.com (I.M.-H.); uqbarita@gmail.com (B.F.-L.); lau.aqu@gmail.com (L.E.J.-A.); mezapilar@yahoo.com (M.d.P.M.-R.); 4 Hospital General de México, Dr. Eduardo Liceaga, Ciudad de Mexico 06720, Mexico; coronel1019@hotmail.com; 5 Department of Human Genetics, University of Utah, Salt Lake City, UT 84112, USA; joselin.hernandez@hsc.utah.edu

**Keywords:** adipokines, inflammation, pregnancy, depression, anxiety, resistin

## Abstract

**Background:** Adipokines secreted by the adipose tissue and placenta play a critical role in regulating metabolic functions that are essential for fetoplacental development and embryonic growth. While adipokines are known to impact a wide range of physiological and pathological conditions, their role in affective disorders during pregnancy remains underexplored. In this study, we aimed to assess the serum levels of distinct adipokines and examine their association with anxiety and comorbid depression in pregnant women. **Methods:** Third-trimester pregnant women with severe anxiety (ANX, *n* = 45) and anxiety plus depressive symptoms (ANX + DEP, *n* = 61) were enrolled in the study, along with healthy control subjects (CTRL, *n* = 33). Participants were classified using the Hamilton Anxiety Rating Scale (HARS) and the Hamilton Depression Rating Scale (HDRS). Serum levels of adiponectin, adipsin, leptin, and resistin were quantified by flow cytometry-based immunoassay. Clinical, biochemical, and demographic parameters were analyzed using ANOVA with a post hoc Tukey test. Pearson bivariate and partial correlations were performed to assess associations between variables. **Results:** Adipokine serum levels were significantly higher in the symptomatic groups (ANX, ANX + DEP) than in the CTRL group (*p* < 0.001). Adiponectin, leptin, and resistin levels positively correlated with anxiety symptoms (HARS, *p* < 0.01). Furthermore, resistin levels showed a strong association with depressive symptoms (HDRS, *p* = 0.001) in the ANX + DEP group, after adjusting all parameters by clinical confounders. **Conclusions:** Our findings revealed that both pro- and anti-inflammatory adipokine levels are elevated in women with affective symptoms during late pregnancy. Pro-inflammatory properties of leptin and resistin may contribute to the severity of anxiety symptoms. Notably, resistin emerges as a key adipokine associated with the expression of depressive symptoms. In addition, adiponectin, acting as an anti-inflammatory mediator, may counteract the inflammatory responses induced by leptin and resistin. These results provide new insights into the role of specific adipocytokine in women with affective disorders during late pregnancy.

## 1. Introduction

Anxiety is a highly prevalent condition in pregnant women [1,2,3]. Estimates of the prevalence of antenatal anxiety range from 15.8% to 25% [1,2], while postpartum anxiety affects between 13 and 31.7% of women [3]. However, other studies report lower rates, with anxiety symptoms affecting 9.5% during pregnancy and 7.6% in the postpartum period [4,5]. A recent meta-analysis reported a prevalence of 37%, with differences according to regions or methods used in research studies [6]. However, certain populations may be more vulnerable to stress, with anxiety symptoms increasing as the due date for labor and delivery approaches [1,4,7]. Interestingly, clinical studies showed that affective symptoms follow a U-shaped curve, displaying the highest symptoms during the first and third trimesters of pregnancy [3,8], finding that stressors represent the single risk factor for the trajectory of depressive and anxiety symptoms in women during pregnancy [1,3].

In many cases, anxiety symptoms are accompanied by depressive symptoms or even a diagnosis of Major Depressive Disorder (MDD). A bidirectional relationship between anxiety and depression has been reported in many scientific studies [9].

Major depressive disorder is a complex and debilitating psychiatric condition [10] responsible for about 10% of global disability (World Health Organization), and is the leading cause of mental impairment worldwide [11].

Meta-analysis studies revealed that the prevalence of antenatal depression ranged from 15 to 65%, globally. Antenatal depression is highly linked to common psychosocial risk factors, particularly in low and middle-income countries, where prenatal depression prevalence is higher than in high-income countries [12,13]. Other reports showed that perinatal depression ranges from 2 to 21% [14,15], but increases to 31% when different self-report score scales are used to screen healthy pregnant population [14,15]. Moreover, prevalence rates for antenatal depression per trimester were estimated at 7.4% (1st trimester), 12.8% (2nd trimester), and 12.0% (3rd trimester) [16,17].

Although prenatal stress and anxiety have been shown to contribute to poor birth and negative neonatal outcomes, these risk factors have not been completely established, and inconsistencies have been found across the literature [18]. Recent studies revealed that the perinatal anxiety symptoms (PAS) rate in a pregnant Mexican population was as high as 21% during pregnancy, with a rate of postpartum anxiety of 18% [19]. These findings are consistent with various reports showing that pregnant women with severe depression and anxiety exhibit elevated levels of inflammatory cytokines than those without these conditions [18,20]. Higher stress scores have been associated with increased levels of pro-inflammatory cytokines (IL-6, TNF-α), alongside reduced levels of the anti-inflammatory marker IL-10 [3]. Other studies have shown significant associations between various serum biomarkers—IL-2, IL-6, IL-10, IL1-B, TNF-α, CRP, CRH, and cortisol—and pregnancy-specific anxiety and depression across all trimesters of gestation [18,21,22].

Inflammation associated with anxiety and depression may, in turn, modulate metabolic systems that are also subject to alteration during pregnancy. This is exemplified by adipokines—metabolic modulators primarily synthesized in adipose tissue—which perform numerous functions in the body, including the regulation of mood [23,24,25]. Notably, cerebrospinal fluid (CSF) levels of several adipokines (e.g., leptin, adiponectin, TNF-α, IL-1β) have been positively correlated with their plasma concentrations, suggesting that circulating adipokines may cross the blood–brain barrier and exert effects on the central nervous system [25,26].

Adipokines have been increasingly studied in the context of affective disorders [23] and have also been associated with various psychological disturbances, most notably eating disorders [27], as well as dementia [28], metabolic syndrome and bipolar depression [29], and sleep disorders [30]. The bidirectional relationship between the HPA axis and adipokines could provide an explanation about the mechanism of this relationship [31].

It has been reported that adiponectin levels were reduced in patients with major depression [32], and preclinical data showed a positive link between circulating adiponectin levels and antidepressant effects [23,32]. Leptin, a key anorexigenic hormone, has also been involved in depression and anxiety. It has been shown that leptin administration in rodents produces potent antidepressant and anxiolytic effects [24,33].

Clinical studies showed positive correlations between leptin serum levels and the severity of depression. Leptin levels in patients with moderate to severe depression were higher than those displaying mild or minimal depression [34]. However, contrasting findings have been reported in reproductive-aged women, where patients with depression and anxiety displayed lower leptin levels compared to healthy controls [35]. Similarly, elevated serum resistin serum levels were detected in adolescents with major depression compared to baseline levels in healthy controls [36].

During pregnancy, the placenta secretes various adipokines such as adiponectin, leptin, resistin, visfatin, and apelin, along with pro-inflammatory cytokines [i.e., TNF-α, IL-6, and IL-1], all of which are involved in regulating adaptive metabolic processes during gestation [37,38]. Elevated baseline serum leptin levels have been reported in pregnant women diagnosed with gestational diabetes mellitus (GDM) [39]. Regarding adiponectin, the consensus states that its levels significantly decrease between the first and third trimesters of pregnancy [40]. In addition, plasma resistin levels at term pregnancy were significantly higher than those in age-matched non-pregnant controls [41].

However, no substantial reports have been documented regarding serum adipokines levels in affective disorders and mood-associated symptoms in pregnant women. To date, only one study has reported an association between the prevalence of psychiatric disorders [MDD, agoraphobia, and generalized anxiety disorder (GAD)] and metabolic markers, including cholesterol, and high- and low-density-related lipoproteins in relation to current suicide risk (CSR) during the first trimester of pregnancy [42].

Despite their known role in metabolic functions during pregnancy, the role of adipokines in mood-related disorders remains poorly understood. However, some studies in non-pregnant women have reported that adiponectin, adipsin, leptin, and resistin may be associated with depressive and anxiety symptoms. Therefore, in this study, we determined the blood levels of key adipokines in pregnant women without metabolic or obstetric complications but exhibiting severe anxiety and comorbid depression.

## 2. Methods

### 2.1. Design of the Study

A quantitative, cross-sectional and analytical study was carried out at the Department of Gynecology & Obstetrics (OB-GYN) in the General Hospital of Mexico (HGM, Dr. Eduardo Liceaga, Mexico City) from February 2015 to October 2017. Third-trimester pregnant women attending the Outpatient Control Unit at the OB-GYN were interviewed by obstetricians to check their health status and course of pregnancy. Patients with no obstetric complications and no acute or chronic illnesses, other than affective disorders, were remitted to the Mental Health Department to receive psychological and psychiatry assessment. At entry, all participants who willingly accepted to participate in the study provided a signed written informed consent before their enrollment into the study. Both Research and Ethics Committees approved the human study enabling the intervention on human subjects (Project ID: D1/14/112/04/072). Sample size of participants included in the study was determined according to convenience, and the statistical number of patients with anxiety and depression were in-coming pregnant women being attended at the control outpatient unit in the OB-GYN department.

All participants with affective symptoms and healthy subjects recruited in the study were Latin-pregnant women with a related economic status (lower middle class) and most of them showed similar activities. Most participants were inhabitants of Mexico City and/or from surrounding states.

Patients in the study underwent third-trimester blood tests, which included complete blood count, blood chemistry (chem-20), lipid profile, hepatic and renal function tests, hormone profile [human chorionic gonadotropin (HCG), estrogen, and progesterone], thyroid profile, and 2D fetal ultrasound. Blood samples were collected at the Hospital’s Central Blood Unit, and biochemical analyses were conducted at the Hospital’s Main Central Lab, the Hospital/Clinical Pharmacology Unit, and the OB-GYN Ultrasound Unit.

### 2.2. Participants

A non-probabilistic sample of pregnant women from 18–35 years-old and coursing a non-complicated third-trimester gestation (28–40 gwks) were invited to participate in the study. At entry, women were interviewed at the HGM/OB-GYN Control Outpatient Unit and both physical and obstetric examinations were uploaded into an Excel DataBase used specifically for patients’ clinical data and variables (Microsoft Office Version 365, USA). During the initial interview, sociodemographic variables (i.e., marital status, education level, working status) and anthropometric measures [height, weight, and body mass index (BMI)] were registered. Participants were then referred to the Mental Health Department for an assessment of their emotional status. The Diagnostic and Statistical Manual of Mental Disorders (DSM-5; American Psychiatric Association, 2015, https://www.psychiatry.org/psychiatrists/practice/dsm) criteria were used to evaluate the presence of any mental disorder other than anxiety or depression, in accordance with the exclusion criteria, for all participants, including healthy controls. Other medical issues related to their pregnancy status were collected and uploaded into the Clinical Database.

The hetero-reported Hamilton Anxiety Rating Scale (HARS) [43] was applied to all participants. This tool is a reliable questionnaire used to assess the intensity of anxiety symptoms. Similarly, the Hamilton Depression Rating Scale (HDRS) [44] is a highly reliable instrument to explore the intensity of depressive symptoms. Scores from evaluated clinician-rated instruments were uploaded into the clinical database.

Patients exhibiting a score ≥ 25 on the HARS scale were considered as individuals with high levels of anxiety symptoms (ANX), whereas patients with a cut-off score < 7 were considered healthy subjects and used as controls (CTRL) in the study [43]. Similarly, pregnant women exhibiting a score > 24 on the HDRS scale were considered patients with higher levels of depressive symptoms (DEP), and patients with a cut-off score < 7 were considered healthy subjects [44].

Women recruited into the study showed no alcohol consumption, no smoking, no drug use or abuse, nor any associated mental pathologies (i.e., bipolar disorder, schizophrenia, psychosis, and neuropsychiatric disorder, including uncontrolled compulsive eating and obsessive disorders). Participants enrolled in the study were not medicated during the initial medical interviews and blood sampling.

The exclusion criteria included subjects taking psychotropic medication or consuming illicit substances of abuse, psychiatric disorders (other than major depression and anxiety diagnosed before or during enrollment of patients), obstetric pathologies, acute or chronic infections, and other medical conditions (i.e., neurological, metabolic, cardiovascular, degenerative, neuroendocrine, immune-related disorders) including assisted conception. Patients with incomplete questionnaires, an absence of or incomplete laboratory tests, inconsistencies in the psychiatric evaluation of mood disorders, missing regular appointments, or the abandonment of the protocol were considered as elimination criteria. After the completion of the hetero-reported clinician-rated questionnaires, patients were remitted to the central lab for blood sampling.

### 2.3. Recruitment Criteria and Clinician-Rated Instruments

Criteria considered for patients’ recruitment were the presence of high anxiety levels following the 14-item Hamilton Anxiety Rating Scale (HARS), which assesses the intensity of anxiety symptoms through a 5-point Likert scale ranging from 0 (no symptom) to 4 (severe anxiety) [43,45]. HARS scores are categorized as follows: mild anxiety (score 7–17), moderate anxiety (score 18–24), and severe anxiety (score ≥ 25). Thus, patients with a final HARS score > 25 were considered pregnant subjects with severe anxiety (ANX). Pregnant women showing scores < 7 were included as healthy controls (CTRL). The anxiety scale used herein has been extensively used to assess the intensity of anxiety-related items, such as subjective feelings, autonomic and somatic symptoms, cognitive functions, and behavioral responses [44].

Depressive symptoms were assessed using the 17-item Hamilton Depression Rating Scale (HDRS) [44]. The Hamilton Rating Scale for Depression is a multidimensional/clinician-rated scale widely used in clinical settings and trials to explore the intensity of depressive symptoms. The intensity of depressive symptoms assessed by the HDRS instrument is based on a Likert scale that assesses the intensity of each item. Thus, higher scores indicate the severity of depression [46].

The Depression Rating Scale was rated as follows: no symptoms (score 0–6), mild depression (score 7–17), moderate depression (score 18–24), and severe depression (score > 24) [44,46]. Pregnant women showing a cut-off score > 24 were considered subjects exhibiting severe depression (DEP). Patients with scores < 7 were rated as healthy controls (CTRL). The depression scale has been widely applied to assess the intensity of depressive mood, suicidal thoughts or actions, insomnia, irritability, fears, somatic symptoms, libido dysfunction, cognitive function, and insight [44]. Moreover, the HDRS is considered a highly useful scale for cognitively impaired patients with difficulty with self-report instruments. Both instruments were shown to be reliable, specific, and sensitive tools [44,46]. Both clinician-rated tools used in the study were validated in the local language (Spanish) [47].

All patients were interviewed by qualified mental health professionals in accordance with the Diagnostic and Statistical Manual of Mental Disorders, Fifth Edition (DSM-5; American Psychiatric Association, 2015, https://www.psychiatry.org/psychiatrists/practice/dsm), to evaluate the presence of any additional psychiatric diagnoses based on the exclusion criteria. The same mental health professionals also administered the Hamilton Depression Rating Scale (HDRS) and provided the Hamilton Anxiety Rating Scale (HARS) to participants. Scores from all clinician-rated instruments were reviewed by the Department of Mental Health.

A total of 139 third-trimester pregnant women were included in the study and according to their affective symptoms, patients were clustered into the following groups: (a) pregnant women exhibiting severe anxiety without depression (ANX, *n* = 45); (b) pregnant women displaying severe anxiety with comorbid depression (ANX + DEP, *n* = 61); (c) healthy control subjects displaying no mood-associated symptoms (CTRL, *n* = 33). Upon completing the clinician-rated scales, all participants (with 8–12 h fasting conditions) were remitted to the clinical laboratory for blood sampling and the quantification of serum analytes.

### 2.4. Blood Sampling

Blood sampling was carried out at the Hospital Central Lab from 7:00 to 9:00 a.m. Blood extraction was performed under aseptic conditions. Briefly, 5.0 mL of venous blood were collected from each patient, in sterile 13 × 100/Vacutainer BD Hemogard Tubes containing Clot Activator/Polymer Gel for serum separation (BD, San Diego, CA, USA). Collected samples were cooled in ice and allowed to clot at 4 °C for 1 h before serum separation. Fresh serum was obtained by centrifuging blood samples at 1600× *g* for 15 min and aliquoted in 2.0 mL Eppendorf tubes. Centrifuged serum vials were then stored at −70 °C until further use.

### 2.5. Quantification of Adipokines in Serum

Serum adipokines (adiponectin, adipsin, leptin, and resistin) were measured using the immunoassay/multiplex bead array, LEGENDplex^TM^ Human Metabolic Panel (4-plex) 100 tests (Cat. 740212, BioLegend, San Diego, CA, USA) following manufacturer’s procedures [20,48]. Briefly, 0.5 mL serum samples were incubated with their specific anti-adipokine antibody-coated beads in a mixed buffer at room temperature for 1.5 h, and a specific fluorochrome was used for each adipokine. Samples were washed with kit buffer and then analyzed in a FACS Aria III flow cytometer (BD Bioscience, Shirley, NY, USA). The LEGENDplexTM Data Analysis Software v 7.0 (Biolegend, San Diego, CA, USA) was used to determine the adipokines’ serum concentrations. The lower limits of detection of each adipokine assayed in the study were human adiponectin (41.4 pg/mL), human adipsin (5.4 pg/mL), human leptin (1.6 pg/mL), and human resistin (1.4 pg/mL). The intra-assay coefficient was <4.0% and the inter-assay covariance was <7.5%.

### 2.6. Statistical Analysis

Adipokine serum levels are shown as scatter plots. Linear regression analysis and (95% CI) of the best-fit slope were calculated for each adipokine. Demographic variables are described in nominal and percentage values (%). Pearson bivariate and partial correlations were performed to assess the associations between biological measures—psychometric scores and clinical variables among the tested groups. Moreover, ANOVA with a post hoc Tukey test was used to detect differences between serum adipokines, clinical measures, and psychometric scores among the study groups. An analysis of covariance was not considered, due to the similarity among adipokines values measured in the study.

All analyses were performed using GraphPad Prism 7 (GraphPad Softwares Inc.: Boston, MA, USA) and SPSS software v.27 (IBM Corp, Armonk, NY, USA). For all the statistical analyses, the *p*-value was set as <0.05.

## 3. Results

### 3.1. Clinical and Demographic Characteristics

Table 1 depicts the clinical variables in non-white Latin, third-trimester pregnant women (*n* = 139) with an average gwk of 34.6 ± 1.2, a mean weight and BMI of 66.2 ± 2.3 (kg) and 27.9 ± 0.5 (kg/m^2^), respectively, and a mean age of 25.5 ± 2.1 years old.

The post hoc Tukey test revealed significant differences in HARS (*p* < 0.001) and HDRS (*p* < 0.05) scores in the symptomatic groups and between affective subjects and controls (*p* < 0.04). Similarly, Table 2 depicts the demographic parameters among the Latin pregnant subjects. As shown, a higher prevalence of cohabitating relations was found in healthy and symptomatic pregnant women (41.4 ± 1.3%) compared to a lower incidence of married (24.7 ± 2.3%) and divorced (23.1 ± 4.8%) subjects. Similarly, regarding the education status, a higher incidence of middle (32.0 ± 3.8%) and high (34.4 ± 5.0%) school education was observed. In addition, a large proportion of pregnant subjects were found to be unemployed (33.2 ± 2.9%) when compared either to the lower prevalence found in women with employment (19.0 ± 2.6%), self-employment (11.8 ± 1.9%), or domestic labors (21.6 ± 2.6%). 

### 3.2. Determination of Adipokines’ Serum Levels

Table 3 shows the serum levels of various adipokines determined in third-trimester pregnant women. As shown, the mean serum levels of adiponectin were significantly higher in the symptomatic groups than in the CTRL group (Tukey test, *p* < 0.001). However, no significant differences were found between the symptomatic groups (Tukey test, *p* = 0.2). Similarly, the mean serum levels of adipsin and leptin were significantly higher in the symptomatic groups compared to the healthy controls (Tukey test, *p* < 0.001), with no significant differences between the anxiety and depressive groups (Tukey test, *p* > 0.31). Regarding resistin, the ANX + DEP group showed the highest serum levels (Tukey test, *p* < 0.001) compared to the CTRL group. The ANX group displayed a nearly marginal increase in resistin levels compared to healthy pregnant group (Tukey test, *p* = 0.06). Moreover, significant differences in the resistin levels were observed between the symptomatic groups (Tukey test, *p* < 0.001).

### 3.3. Scatter Plots of Serum Adipokines

**Figure 1:**
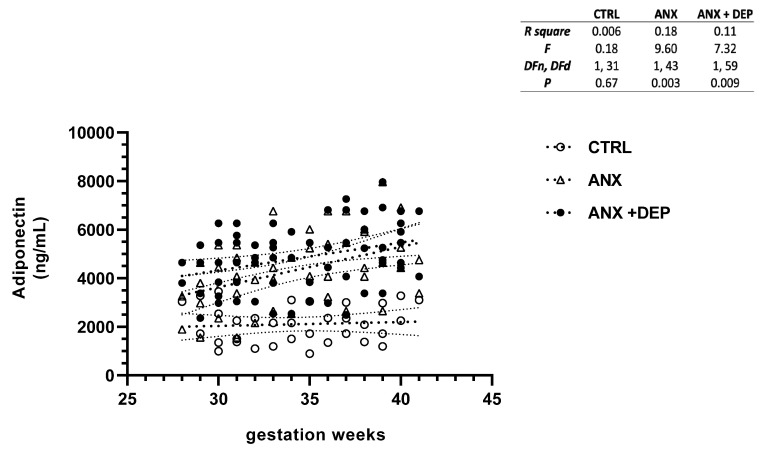
**Adiponectin levels in pregnant women with anxiety and depression.** The figure depicts serum adiponectin levels across the gwk timeline during the third trimester in pregnant women, grouped into control (CTRL), anxiety (ANX), and anxiety with comorbid depression (ANX + DEP) categories. The inset describes the linear regression analysis of the adipokine levels as measured by flow cytometry. Abbreviations: CTRL, control, ANX, anxiety; ANX + DEP, anxiety plus comorbid depression. Data were calculated using GraphPad v.7. Significant differences were established at *p* < 0.05.

**Figure 2:**
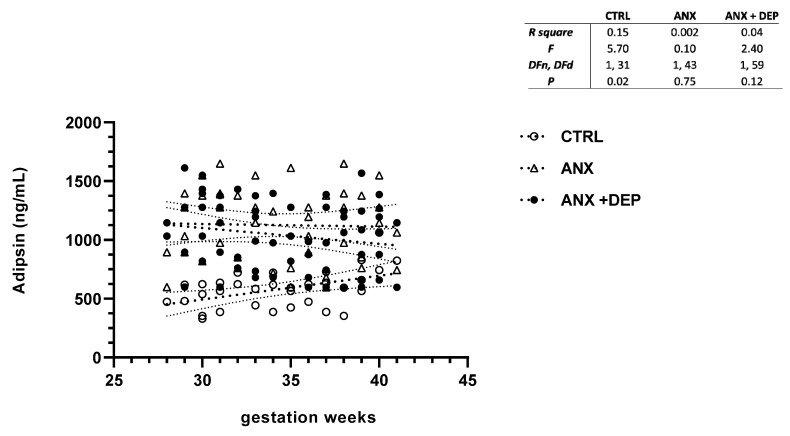
**Adipsin levels in pregnant women with anxiety and depression.** The figure depicts serum adipsin levels across the gwk timeline during the third trimester in pregnant women, grouped into control (CTRL), anxiety (ANX), and anxiety with comorbid depression (ANX + DEP) categories. The inset describes the linear regression analysis of the adipokine levels as measured by flow cytometry. Abbreviations: CTRL, control, ANX, anxiety; ANX + DEP, anxiety plus comorbid depression. Data were calculated using GraphPad v.7. Significant differences were established at *p* < 0.05.

**Figure 3:**
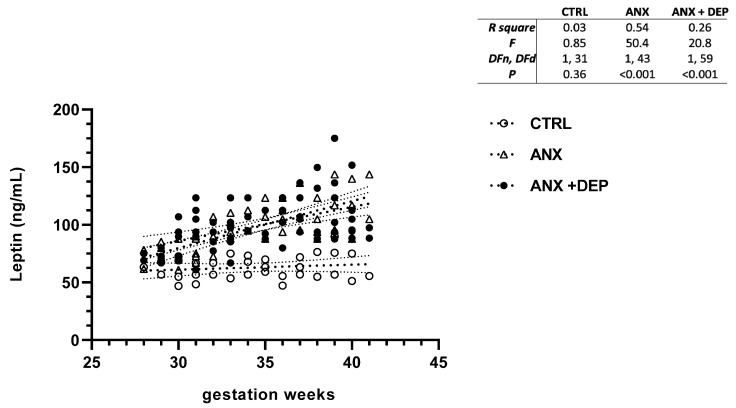
**Leptin levels in pregnant women with anxiety and depression.** The figure depicts serum leptin levels across the gwk timeline during the third trimester in pregnant women, grouped into CTRL, ANX, and ANX + DEP categories. The inset describes the linear regression analysis of the adipokine levels as measured by flow cytometry. Abbreviations: CTRL, control, ANX, anxiety; ANX + DEP, anxiety plus comorbid depression. Data were calculated using GraphPad v.7. Significant differences were established at *p* < 0.05.

**Figure 4:**
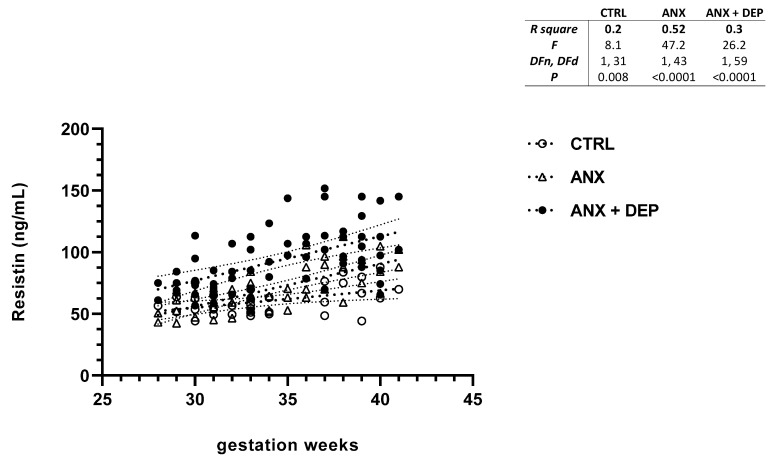
**Resistin levels in pregnant women with anxiety and depression.** The figure depicts serum resistin levels across the gwk timeline during the third trimester in pregnant women, grouped into CTRL, ANX, and ANX + DEP categories. The inset describes the linear regression analysis of the adipokine levels as measured by flow cytometry. Abbreviations: CTRL, control, ANX, anxiety; ANX + DEP, anxiety plus comorbid depression. Data were calculated using GraphPad v.7. Significant differences were established at *p* < 0.05.

Figure 1, Figure 2, Figure 3 and Figure 4 depict the linear regression of scatter plots of serum levels of adiponectin, adipsin, leptin, and resistin among the study groups. As shown, higher serum levels of adiponectin were increased as the gwk timeline period was approaching the estimated labor due date in the symptomatic groups (ANX, ANX + DEP), as compared to the linear, non-dispersed adipokine levels detected in the CTRL group. However, a higher dispersion of serum adiponectin levels was detected in the ANX + DEP group compared to the anxiety and control groups (Figure 1, scatter plot). Furthermore, significant differences in the linear regression of best-fit slope analysis for adiponectin were observed in the symptomatic groups (95% CI, R^2^ < 0.2, *p* < 0.009) as compared to the CTRL group (95% CI, R^2^ > 0.1, *p* < 0.01) (Figure 1, inset).

In the same way, the scatter plot for adipsin showed slight decreases in this adipokine serum levels between the symptomatic groups (ANX, ANX + DEP) along the gwk timeline period. Conversely, higher adipsin serum levels were observed in the CTRL group (Figure 2, scatter plot). As shown, no differences in the linear regression best-fit slope analysis for adipsin were found in the anxious and depressive groups (95% CI, R^2^ > 0.04, *p* > 0.12). However, a significant difference in the calculated linear regression slope value for the adipokine was observed in the CTRL group (95% CI, R^2^ = 0.15, *p* = 0.02) (Figure 2, inset).

Similarly, the scatter plot for leptin revealed significant increases in this serum adipokine levels in the emotionally affected pregnant population (ANX, ANX + DEP). As shown, a high dispersion of leptin serum levels was detected in the symptomatic groups as the gwk timeline period approached the estimated labor due date (Figure 3, scatter plot). Significant differences were observed in the linear regression best-fit slope analysis for this adipokine in the symptomatic groups (ANX, R^2^ = 0.54, *p* < 0.001; ANX + DEP, R^2^ = 0.26, *p* < 0.001) compared to the CTRL (R2 = 0.03 *p* = 0.36). No significant differences in the estimated linear regression slope value for leptin were found in the CTRL group (95% CI, R^2^ = 0.15, *p* = 0.02 (Figure 3, inset).

In addition, the resistin scatter plot showed an increase in serum levels of this adipokine in both anxious (ANX) and depressive (ANX + DEP) groups as the gwk timeline period approached full-term pregnancy. Moreover, the scatter plot showed a high dispersion of resistin levels in the ANX + DEP group, compared to the ANX group. Nonetheless, slight increases in resistin levels were observed in the CTRL group (Figure 4, scatter plot). Significant differences were found in the linear regression best-fit slope analysis for resistin in the symptomatic groups (ANX, R^2^ = 0.52, *p* < 0.001; ANX + DEP, R^2^ = 0.30, *p* < 0.001) and the CTRL (R^2^ = 0.20 *p* = 0.008) (Figure 4, inset).

### 3.4. Bivariate Correlations

Table 4A,B illustrate the bivariate correlations between serum adipokine levels and clinical measures among the study groups. As shown, significant higher correlations were observed between leptin, resistin, and anxiety symptoms (HARS scores) in the symptomatic groups (*p* < 0.004). Significant associations between adiponectin levels and HARS scores were observed in the ANX group (*p* = 0.02) (Table 4A). Such adipokines showed significant correlations with gwk (*p* < 0.01), weight (*p* < 0.02), and BMI (*p* = 0.02) in the affective symptomatic groups (Table 4A,B). Moreover, the tested groups found significant correlations between adiponectin, leptin, and resistin serum levels (*p* < 0.01). Interestingly, a significant high association was found between resistin serum levels and depressive symptoms (HDRS scores) in the ANX + DEP group (*p* = 0.001) (Table 4B), suggesting that this adipokine should be related to the affective symptoms in the study groups. 

Moreover, the post hoc Tukey test showed significant differences between resistin and leptin serum levels (*p* < 0.001), resistin and adiponectin (*p* < 0.01). 

### 3.5. Partial Correlations Adjusted by Confounders

Table 5 depicts the Pearson partial correlations after controlling biochemical and clinical measures by individual confounders (BMI, gwk) in the symptomatic groups. After controlling all parameters for gwk, serum levels of adiponectin, leptin, and resistin displayed significantly high correlations with anxious symptoms (HARS, *p* = 0.001). Similarly, after controlling our variables for BMI, leptin and resistin displayed significantly high correlations with high levels of anxiety (HARS, *p* < 0.005), while adiponectin showed also an association with anxiety symptoms (HARS, *p* = 0.01). Only resistin displayed highly significant correlations with HDRS scores in the symptomatic groups after variables were adjusted by clinical confounders (*p* = 0.001). Additionally, significant positive correlations were observed between these adipokines. (*p* < 0.03) (Table 5).

## 4. Discussion

Several adipokines (i.e., adiponectin, leptin, resistin, visfatin, and apelin, among other mediators) are secreted from the fetoplacental interface into the maternal circulation [38]. These factors play a crucial role in modulating and driving metabolic processes and adaptive conditions that induce the most favorable and stable environment for blastocyst implantation, placental development, and embryonic growth during early stages of gestation [37,49].

Several adipokines (leptin, adiponectin, resistin, visfatin, chemerin, vaspin, apelin, omentin) that are released from adipose tissue are also secreted by macrophages, neutrophils, basophils, and mast cells. The pro-inflammatory adipokines (e.g., leptin, resistin) are overproduced with increasing adiposity, while adipokines with anti-inflammatory properties (e.g., adiponectin, omentin) are decreased [50]. Leptin is mainly secreted from adipose tissue and to a lesser extent from T cells, and its secretion is mostly regulated by sex steroids, growth hormones, and inflammatory cytokines (IL-1, TNF-α) [51]. The resistin (Rstn) gene and its protein are mainly expressed in white adipocytes and blood cells in rodents [46]. However, peripheral blood mononuclear cells, macrophages, and bone marrow cells were reported to be primary source of circulating resistin in humans [25,37].

Adiponectin is one of the most studied adipokines in pregnancy. It is secreted from multiple cells (i.e., osteoblasts, liver, parenchyma cells, myocytes, epithelial cells, placenta) [50,52] and exerts pleiotropic functions ( e.g., energy metabolism; antioxidant, anti-inflammatory, anti-atherosclerotic effects; glucose homeostasis; insulin sensitivity) [37,38,50]. Previous studies showed that adiponectin anti-inflammatory effects are mediated by the suppression of M1 macrophages, which promote pro-inflammatory factors and induce insulin resistance, stimulating M2 cells, enhancing the release of anti-inflammatory factors, such as IL-10, reducing the secretion of IFN-γ, IL-6, and TNF-α [53]. Moreover, adiponectin binding its cognate surface receptors (AdipoR1, AdipoR2) suppresses the IKK-NF-κB-PTEN signaling pathway, reducing IL-6 and TNF-α cell expression [54,55]. These results supported the findings about the negative correlation observed between adiponectin and IL-6 and the high-sensitivity C-reactive protein (CRP) [56] observed in affective disorders.

Leptin exerts wide and varied immune-related short-term responses by releasing several immune mediators [i.e., proinflammatory cytokines (TNF-α, IL-1b, IL-6, IL-18), chemokines (CCL, CCL3) leukotrienes (cysLTs), LTB4, and prostaglandin E2] that modulate the course of inflammation in different aspects [57,58] and stimulate the migration and infiltration of monocytes and neutrophils in sites of tissue injury [59,60]. Furthermore, several pieces of evidence support the idea that leptin contributes to the amplification of the inflammatory process by inhibiting the production of IL-10 in NK [61] and in DC [62] cells, in addition to decreasing the TGF-β production by macrophages (MΦ) [63].

The pro-inflammatory responses of leptin could be associated with the anxiety-related behaviors [64] promoted by this adipokine in the hypothalamus (lateral arcuate nucleus and paraventricular nucleus) [65,66], together with other peptide hormones (ghrelin) [67]. Similarly, leptin was found to modulate depressive-like behaviors [68] driven by same hypothalamic nuclei described above [65,66]. Furthermore, leptin receptor is expressed in dopamine projecting neurons in the that have been implicated in food-seeking behavior [66].

However, knockdown experiments on the Leptin receptor on dopaminergic neurons of the ventral tegmental area did not show any influence in the motivational food-seeking behavior, but rather increased anxiety-related behavior [66]. Thus, leptin’s role in modulating anxiety-related behaviors at specific neural targets [65,69] may be mediated, at least in part, by the upregulation of sympathetic nervous system (SNS) activity commonly observed in anxiety states.

Furthermore, the increased leptin levels are crucial in mediating anxiolytic responses via the activation of its receptor related signaling pathways in the hypothalamus [65], and in dopaminergic neurons of the ventral tegmental area in response to anxiogenic stress responses [66,70]. Moreover, recent studies showed that the increased leptin plasma levels appear to represent an indirect mediator of clinical depression status and “somatic anxiety” symptoms in depressive individuals [71].

Hitherto, our findings about the increased leptin levels in symptomatic pregnant women suggest that this adipokine exerts a key role in modulating stress-inducing high levels (somatic) of anxiety symptoms in response to anxiogenic stress responses in vulnerable pregnant women. Similarly, pregnant women exhibiting high levels of anxiogenic responses could affect the serum levels, activating the adipokine-related signaling pathway on target neurons in the brain. Such a hypothesis is supported by findings showing that leptin plasma levels were positively correlated with BMI in reproductive women with anxiety states [35,72]. It is worth mentioning that increased circulating leptin levels were reported in individuals with acute stress displaying normal weight and BMI [73], supporting, thus, that leptin may enhance anxiety symptoms in vulnerable pregnant women with normal baseline weight and BMI measures, as shown herein.

Resistin was found to induce a significant inhibition of dopamine and noradrenaline synthesis in the hypothalamus [74], suggesting that the activation of the resistin cell-signaling system on target neurons [24] and on Th1-immune cells [75] exerts an influence on catecholaminergic neural pathways [74] and, hence, on the neural mechanisms implicated in major depression [63,64]. In line with this, our results posit that stress-inducing anxiety and depression observed in pregnant women could be due to the increased circulating levels of both leptin and resistin, in addition to other immune biomarkers shown to be increased in pregnant subjects with major depression [18]. However, impinging stressors and anxiogenic responses in vulnerable pregnant women may impact the increased serum levels of both adipokines. In support of this idea, recent studies showed that increased resistin serum levels in major depression subjects correlated with high scores for depression (HAM-D) [71].

Adiponectin levels correlated with leptin and resistin serum levels in our study. Leptin and adiponectin have opposing reactions. The former upregulates pro-inflammatory factors such as TNF-α and IL-6, while the latter down-regulates these immune mediators [76]. Thus, the decreased adiponectin levels bring about several physiological changes such as insulin resistance, cardiovascular dysfunction, and inflammation-related diseases [77]. Moreover, it has been shown that patients with metabolic diseases like T2D, obesity, and chronic inflammation displayed low serum adiponectin levels [77].

In contrast, patients with autoimmune diseases and chronic inflammation showed high serum adiponectin levels [77].

A plausible explanation for such discrepancy may rely on the multi-faceted role of adiponectin and its isoforms, which activate distinct signaling systems [50]. Thus, the increase in serum adiponectin levels suggests that its anti-inflammatory activity may counteract or offset the pro-inflammatory processes promoted by leptin, resistin, and other inflammatory immune mediators in pregnant individuals experiencing severe anxiety [22] and depression [20,21]. For instance, the leptin–adiponectin ratio has been suggested as a potential valuable marker to assess the increase or decrease in inflammatory responses in key roles in fetal–maternal metabolism, fetal–maternal communication, and gestation [78]. Thus, the leptin–adiponectin and/or resistin–adiponectin ratios could represent plausible potential biomarkers for depression and anxiety, in addition to the Th1–Th2 ratio, which provides a relationship between pro-inflammatory and anti-inflammatory activity mediated by interleukines during pregnancy, as previously reported [20].

In summary, our results demonstrate that elevated serum levels of inflammatory adipokines are significantly associated with anxiety and depressive symptoms in pregnant women. Notably, anthropometric measures did not appear to contribute to the clinical manifestation of both affective states in the symptomatic groups. The present study provides exciting highlights about the potential role of pro-inflammatory adipokines in mood disturbances during pregnancy, suggesting that the dysregulation of peripheral adipocytokines influences neural pathways involved in the development of mood-associated disorders.

## 5. Conclusions

Our findings reveal that pregnant women experiencing perinatal anxiety and depression exhibited elevated levels of adiponectin, leptin, and resistin levels compared to healthy pregnant subjects. Moreover, leptin and resistin levels showed weak correlations with baseline anthropometric measures, suggesting that their alterations are more linked to affected disorders than to body composition. Notably, resistin levels showed a strong association with depressive symptoms. Thus, our study adds valuable information about the inflammatory and anti-inflammatory profiles of adipocytokines in pregnant women with affective disorders. It is worth noting that leptin serum levels appears to be increased in perinatal depression due to the presence of the fetoplacental unit, whose secretion from the placenta stimulates the migration and infiltration of immune cells (see text in discussion), and, as the semi-allograft tissue considered as a “varied version” of injury tissue, this may respond by enhancing an inflammatory response, evoking the release of pro-inflammatory biomarkers from the invading trophoblast and, hence, increasing the serum levels of leptin compared to non-pregnant subjects, which contributes to an increased inflammatory response in perinatal depression.

## 6. Perspectives

Our findings about the increased circulating levels of both leptin and resistin in pregnant women with anxiety and depression posit that these two important adipocytokines (released from adipocytes or the brain) dysregulate the HPA axis bioactivity, at distinct levels, leading to hypercortisolemia [31]. Pharmacological approaches using escitalopram and other SSRIs were found to reduce (IL)-6 levels and TNF-α levels in patients remitted from depression, and it is quite interesting to note that low levels of IL-1β and leptin levels predicted remission in association with SSRI treatment [79] in addition to improving the aberrant HPA dysfunction to baseline activity [80]. Thus, antidepressant treatment for mood disorders seems an appropriate approach to reduce the altered levels of pro-inflammatory cytokines and adipokines among several other mediators [81], in addition to a strict adherence to psychological intervention [e.g., Cognitive–Behavioral Therapy (CBT)]. Nonetheless, a wide and vast literature has emerged over the past decades, describing the use of multimodal administration of novel and classical pharmacological agents to provide a faster and optimal recovery from depression.

## 7. Limitations

This study has several limitations. First, the cross-sectional nature of our design limits the ability to draw causal conclusions, as it is based on a single time-point measurement. This type of methodological approach does not allow us to determine the directionality of the observed associations, and we cannot conclude whether stress responses lead to elevated adipokine levels or are a consequence of them. Additionally, the study captures data only during late pregnancy. A longitudinal study from early pregnancy to the postpartum period would be valuable for detecting trajectories of adipokine profiles and related metabolic changes.

Second, data were collected exclusively from women with severe anxiety and depression, without considering milder symptomatology. Therefore, the generalizability of our results is limited, as they apply only to pregnant women with high scores on depression and anxiety scales.

Finally, our sample size was small due to the difficulty in recruiting participants who met all the inclusion criteria. Future longitudinal research with a larger sample may provide more robust evidence to validate and expand upon our findings.

## Figures and Tables

**Table 1:** Clinical measures among the study groups.

Subjects *n* = 139	CTRL *n* = 33	ANX *n* = 45	AND + DEP *n* = 61	
**CLINICAL**				**Tukey Test**
**(Parameters)**	**mean (SD)**	**mean (SD)**	**mean (SD)**	***p* value**
**age (years)**	27.6 (7.6)	25.5 (5.8)	25.2 (6.3)	0.19 ^a^
**gwk**	34.9 (3.7)	34.8 (4.0)	34.8 (3.6)	0.96 ^a^
**weight (Kg)**	68.2 (9.8)	66.5 (8.9)	67.4 (11.6)	0.75 ^a^
**BMI (Kg/m** **^2^)**	28.1 (3.7)	28.1 (3.5)	27.7 (4.0)	0.82 ^a^
**HARS (score)**	3.0 (0.6)	24.1 (5.5)	28.1 (5.3)	<0.001 ^b,c,d^
**HDRS (score)**	4.5 (0.8)	6.9 (1.0)	32.3 (7.1)	<0.04 ^b,c,d^
**gestation weeks**	***n* (%)**	***n* (%)**	***n* (%)**	
**27–29**	4 (12.2)	6 (13.3)	7 (11.5)	
**30–32**	8 (24.2)	11 (24.4)	16 (26.6)	
**33–35**	8 (24.2)	8 (17.8)	12 (19.7)	
**36–38**	7 (21.2)	12 (26.7)	12 (19.7)	
**39–41**	6 (18.2)	8 (17.8)	14 (22.9)	

The post hoc Tukey test was used to detect statistical differences in the clinical measures among the study groups. Data are expressed as the mean ± SD. ^a^ Comparisons among the study groups. ^b^ Comparisons between ANX and CTRL groups. ^c^ Comparisons between ANX + DEP and CTRL groups. ^d^ Comparison between ANX + DEP and ANX groups. Abbreviations: CTRL, control; ANX, anxiety; ANX + DEP, anxiety plus comorbid depression; HDRS, Hamilton Depression Rating Scale; HARS, Hamilton Anxiety Rating Scale; BMI, Body Mass Index; gwk, gestational weeks. Data were calculated using GraphPad Prism v.7 and SPSS v.25. Significant differences were established at *p* < 0.05.

**Table 2:** Sociodemographic parameters among the study groups.

Demographic Parameters	CTRL	ANX	ANX + DEP
Participants	*n* = 33	*n* = 45	*n* = 61
**Marital status**	***n* (%)**	***n* (%)**	***n* (%)**
Never Married	4 (12.2)	6 (13.3)	8 (13.1)
Married	7 (21.2)	12 (26.7)	16 (26.2)
Divorced	10 (30.3)	8 (17.8)	13 (21.3)
Cohabiting	17 (42.5)	19 (42.2)	24 (39.4)
**Education level**	***n* (%)**	***n* (%)**	***n* (%)**
Elementary school	0 (0)	4 (8.9)	9 (14.8)
Middle school	9 (27.3)	14 (31.1)	23 (37.7)
High school	13 (39.3)	17 (37.8)	16 (26.2)
Bachelor’s degree	8 (24.2)	7 (15.5)	8 (13.1)
Postgraduate	3 (9.1)	3 (6.7)	4 (6.6)
Technician degree	0 (0)	0 (0)	1 (1.6)
**Working status**	***n* (%)**	***n* (%)**	***n* (%)**
Employee	5 (15.1)	10 (22.2)	12 (19.7)
Self-Employed	3 (9.0)	6 (13.3)	8 (13.1)
Unemployed	12 (36.3)	13 (28.9)	21 (34.4)
Home labor	8 (24.2)	8 (17.8)	14 (23.0)
Commerce	2 (6.0)	6 (13.3)	6 (9.8)
Profession	3 (9.0)	2 (4.5)	0 (0)

Demographic parameters among the study groups are depicted in nominal and percent values. Abbreviations: CTRL, control; ANX, anxiety; ANX + DEP, anxiety plus comorbid depression.

**Table 3:** Determination of serum adipokine levels among the study groups.

Adiponectin	Group	Mean (ng/mL)	SD	Tukey Test
				*p* Value
	**CTRL**	2107.8	775.4	
	**ANX ^a^**	4401.6	1547.6	<0.001 *
	**ANX + DEP ^b^**	4845.7	1351.7	<0.001 *
	**ANX vs ANX + DEP**			0.24
				**Tukey test**
**Adipsin**	**group**	**mean (ng/mL)**	**SD**	***p* value**
	**CTRL**	581.2	159.9	
	**ANX ^a^**	1120.1	308.8	<0.001 *
	**ANX + DEP ^b^**	1038.9	290	<0.001 *
	**ANX vs ANX + DEP**			0.31
				**Tukey test**
**Leptin**	**group**	**mean (ng/mL)**	**SD**	***p* value**
	**CTRL**	62.7	9.8	
	**ANX ^a^**	96.7	21.7	<0.001 *
	**ANX + DEP ^b^**	99.8	22.6	<0.001 *
	**ANX vs ANX + DEP**			0.71
				**Tukey test**
**Resistin**	**group**	**mean (ng/mL)**	**SD**	***p* value**
	**CTRL**	60.6	11.5	
	**ANX ^a^**	71.3	18.5	0.06
	**ANX + DEP ^b^**	93.3	25.7	<0.001 *
	**ANX vs ANX + DEP**			<0.001 *

GraphPad Prism v.7 was used to calculate the mean and SD values of each adipokine level measured by flow cytometry. SPSS v.25 was used to establish the statistical differences of the adipokines among the study groups using ANOVA with post hoc Tukey test. Data are expressed as the mean ± SD. ^a^ Comparisons between ANX and CTRL groups. ^b^ Comparisons between ANX + DEP and CTRL groups. (*) Values were significant different at *p* < 0.01. Abbreviations: CTRL, control; ANX, anxiety; ANX + DEP, anxiety plus comorbid depression. Significant differences were established at *p* < 0.05.

**Table 4:** Bivariate correlations between serum adipokine levels and clinical measures in the anxiety group.

**(A)**									
**ANX**	**Adipokine**	**gwk**	**Weight**	**BMI**	**HARS**		**Adiponectin**	**Leptin**	**Resistin**
	**A** **d** **iponectin**								
	Corr.	0.47 **	—	—	0.33 *		—	—	0.40 *
	Sig.	0.001	—	—	0.02		—	—	0.006
	**Le** **p** **tin**								
	Corr.	0.67 **	0.34 *	0.33 *	0.48 **		—	—	0.38 **
	Sig.	<0.001	0.02	0.02	0.003		—	—	0.009
	**R** **esistin**								
	Corr.	0.72 **	—	—	0.46 **		0.40 **	0.38 **	—
	Sig.	<0.001	—	—	0.001		0.006	0.009	—
**(B)**									
**ANX+ DEP**	**Adipokine**	**g** **wk**	**w** **e** **ight**	**BMI**	**H** **ARS**	**HD** **R** **S**	**Adiponectin**	**Le** **ptin**	**R** **e** **sistin**
	**Adiponectin**								
	Corr.	0.35 *	0.34 *	—	0.28	—	—	0.40 **	0.38*
	Sig.	0.01	0.02	—	0.06	—	—	0.006	0.01
	**Le** **p** **tin**								
	Corr.	0.46 **	0.44 **	0.31 *	0.52 **	—	0.40 **	—	0.43 **
	Sig.	0.001	0.002	0.02	<0.001	—	0.006	—	0.002
	**R** **e** **sistin**								
	Corr.	0.54 **	—	0.34 *	0.41 **	0.46 **	0.38 *	0.43 **	—
	Sig.	<0.001	—	0.02	0.004	0.001	0.01	0.002	—

SSPS software v.25.0 was used to determine the Pearson bivariate correlations between clinical parameters and serum adipokine levels among the study groups. (*) Values were significant different at *p* < 0.05. (**) Values were significant different at *p* < 0.01. Abbreviations: CTRL, control; ANX, anxiety; ANX + DEP, anxiety plus comorbid depression; HDRS, Hamilton Depression Rating Scale; HARS, Hamilton Anxiety Rating Scale; BMI, Body Mass Index; gwk, gestational weeks; Corr., correlation; Sig., significance. Significant differences were established at *p* < 0.05.

**Table 5:** Partial correlations between serum adipokine levels and clinical measures.

**Group**	**Controled for**	**Adipokine**	**Weight**	**BMI**	**HARS**	**HDRS**	**Adiponectin**	**Leptin**	**Resistin**
**ANX**	**gwk**	**Adiponectin**							
**ANX + DEP**		Corr.	—	—	0.33 **	—	—	0.47 **	0.31 **
		Sig.	—	—	0.001	—	—	<0.001	0.001
		**Leptin**							
		Corr.	0.21 *	0.23 *	0.34 **	—	0.47 **	—	0.49 **
		Sig.	0.03	0.01	0.001	—	<0.001	—	<0.001
		**Resistin**							
		Corr.	—	0.21	0.30 *	0.48 **	0.31 **	0.49 **	—
		Sig.	—	0.05	0.001	<0.001	0.001	<0.001	—
**Group**	**Controled for**	**Adipokine**	**gwk**	**Weight**	**HARS**	**HDRS**	**Adiponectin**	**Leptin**	**Resistin**
**ANX**	**BMI**	**Adiponectin**							
**ANX + DEP**		Corr.	—	—	0.23 *	—	—	0.49 **	0.32 **
		Sig.	—	—	0.01	—	—	<0.001	0.001
		**Leptin**							
		Corr.	0.27 **	0.29 **	0.25 **	—	0.49 **	—	0.23 *
		Sig.	0.003	0.007	0.005	—	<0.001	—	0.03
		**Resistin**							
		Corr.	0.34 **	—	0.45 **	0.24 **	0.32 **	0.23 *	—
		Sig.	0.002	—	<0.001	0.009	0.001	0.03	—

SSPS software v.25.0 was used to determine the Pearson partial correlations between clinical measures and serum adipokines adjusted by confounders in the symptomatic groups. (*) Values were significant different at *p* < 0.05. (**) Values were significant different at *p* < 0.01. Abbreviations: ANX, anxiety; ANX + DEP, anxiety plus comorbid depression; HDRS, Hamilton Depression Rating Scale; HARS, Hamilton Anxiety Rating Scale; BMI, Body Mass Index; Corr., correlation; Sig., significance. Significant differences were established at *p* < 0.05. See text for more details.

## Data Availability

Data supporting reported results can be found in published papers and can be found at the following links: https://pubmed.ncbi.nlm.nih.gov/30943938/ (accessed on 3 April 2019); https://pubmed.ncbi.nlm.nih.gov/?term=chemokines++pregnancy+anxiety+depression (accessed on 4 December 2021); https://pubmed.ncbi.nlm.nih.gov/32758184/ (accessed on 5 August 2020).
